# Bingeing as an ADHD-related strategy: a qualitative study of experiences of Neurodivergent and potentially Neurodivergent adults with bulimic-spectrum eating disorders

**DOI:** 10.1007/s40519-025-01804-6

**Published:** 2025-12-02

**Authors:** Lauren Makin, Adia Meyer, Dimitri Chubinidze, Valeria Mondelli, Kate Tchanturia

**Affiliations:** 1 PO59, Department of Psychological Medicine, King’s College London, Institute of Psychiatry, Psychology & Neuroscience, 16 De Crespigny Park, London, SE5 8AF UK; 2 National Institute for Health and Care Research (NIHR) Maudsley Biomedical Research Centre at South London and Maudsley NHS Foundation Trust and King’s College London, London, UK; 3 Centre for Research in Eating and Weight Disorders (CREW), Department of Psychological Medicine, King’s College London, London, UK; 4 Department of Eating Disorders, South London and Maudsley NHS Foundation Trust, London, UK; 5 Ilia State University, Tbilisi, Georgia

**Keywords:** Bulimia nervosa, Autism, ADHD, Neurodivergence, Eating disorders, Qualitative research

## Abstract

**Purpose:**

ADHD and Autism are overrepresented in adults with bulimic-spectrum eating disorders (EDs) and are associated with unique underlying mechanisms and poorer treatment outcomes. This qualitative study explores how Neurodivergent and potentially Neurodivergent individuals with bulimic-spectrum EDs make sense of their (potential) Neurodivergence, its impact on their ED, and their treatment needs.

**Methods:**

Sixteen adults with bulimic-spectrum EDs who either self-reported a diagnosis of ADHD and/or Autism or scored highly on screeners (ASRS-Screener > 3; AQ-10 > 5) were interviewed. Data was analysed using reflexive thematic analysis, with cross-group comparisons between ADHD-only and ADHD + Autism presentations. Reflexivity was strengthened through *critical friend* discussions, and *member reflections*.

**Results:**

We developed four themes and 12 sub-themes: 1. *Difficulty making sense of potential Neurodivergence*: participants expressed mixed feelings about identifying as Neurodivergent. While some found the label helpful, others felt uncertain about whether they were Neurodivergent or had concerns around stigma. Participants struggled to distinguish features of Neurodivergence from those of their ED. 2. *Bingeing as ADHD self-regulation*: bingeing was used to manage emotional overwhelm or under-stimulation linked to ADHD, and often became compulsive over time. 3. *Restriction shaped by Autistic traits*: restriction was associated with interoceptive and exteroceptive sensory differences, preference for sameness, and social disconnect, particularly among those with co-occurring Autism. 4. *Balancing personalised and structured care*: participants wanted flexible, personalised care that also provided structure to support recovery.

**Conclusions:**

ADHD and Autistic traits may influence bulimic-spectrum EDs in distinct ways. Helping Neurodivergent individuals and clinicians understand these connections can guide personalised treatment priorities and adaptations, improving treatment engagement and outcomes for Neurodivergent individuals.

*Level of Evidence*: Level IV, qualitative exploratory study.

## Introduction

‘Neurodivergence’ is an umbrella term describing neurotypes that differ from what is typically considered the societal ‘norm’ [1]. It stems from the Neurodiversity paradigm, which frames such differences as natural variations in human cognition rather than deficits [2]. ADHD and Autism are common forms of Neurodivergence that often co-occur and are associated with differences in executive functioning, sensory processing, and communication [3, 4]. Though Neurodivergence can refer to a range of neurotypes (e.g. Dyslexia, Intellectual Disability, Tourette’s Syndrome) [1, 5, 6], ADHD and Autism are the neurotypes most associated with eating disorders (EDs) [6]. They are overrepresented in adult populations with bulimic-spectrum EDs [7, 8], and are associated with distinct ED mechanisms and treatment needs [9–11].

Bulimic-spectrum EDs include *bulimia nervosa*, characterised by recurrent episodes of binge eating followed by compensatory behaviours, such as purging or fasting; *atypical bulimia nervosa*, a sub-threshold form of bulimia nervosa where individuals still display bingeing and compensatory behaviours but do not meet full diagnostic criteria (e.g. lower frequency or limited duration); and *purging disorder*, which involves recurrent purging behaviours (e.g. self-inducted vomiting, laxative, or diuretic misuse) without the presence of binge eating episodes [3].

### ADHD in EDs

ADHD is highly prevalent in bulimic-spectrum ED populations [12]. 15% of adults with bulimia nervosa meet diagnostic criteria for ADHD [13], compared to 3% of the general population [14]. Furthermore, higher percentages of patients with binge-purge subtypes of anorexia nervosa (35%) or ED not otherwise specified (31%) screened positively for ADHD compared to patients with restrictive subtypes (18%, 26%) [8].

Proposed mechanisms linking ADHD and bulimic-spectrum EDs include impulsivity and emotion regulation differences (especially negative urgency), and reward sensitivity, such as heightened drive for immediate rewards. All of these are thought to contribute to binge eating [15–19]. Evidence from a qualitative study on possibly ADHD (and Autistic) adults with binge eating pathology supports these [20]. Participants reported that stimulation seeking drove bingeing as a means of procrastination or coping with boredom; emotional overwhelm prompted bingeing for comfort; and impulsivity further amplified these responses. However, they also reported that other ADHD-related factors, such as long-acting medication and hyper-focusing often led participants to forget to eat. Executive functioning differences also made food shopping and preparation difficult. Some studies also suggest that bulimic-spectrum EDs may exacerbate ADHD traits [8, 15]. However, most existing research has focused on binge eating rather than clinical bulimic-spectrum ED populations. Purging behaviours and patients’ own interpretations of the ADHD-ED link remain underexplored [7], limiting the clinical relevance of current findings for both patients and clinicians.

Furthermore, ADHD is associated with higher treatment dropout and poorer outcomes in ED services [11], yet no ADHD-specific adaptions currently exist for ED treatment [7]. Possibly ADHD (and Autistic) adults with binge eating pathology described needing strategies to help them remember to eat regularly, make food preparation easier, and meet their stimulation needs without relying on food [20]. They also wanted short, regular, and interactive treatment sessions and peer support from other Neurodivergent individuals [20].

### Autism in EDs

Autism is also elevated in patients with bulimic-spectrum EDs; Two previous studies have found Autistic traits to be significantly elevated in patients with bulimia nervosa compared to healthy controls [21, 22]. Despite this, research into Autism and EDs has predominantly focused on restrictive EDs [7]. Brede and colleagues [10] developed a theoretical model for Autism and restrictive EDs, identifying Autistic traits—such as emotional, sensory, and social differences, and preferences for routine—as perceived contributors to restrictive eating. Kinnaird and colleagues [23] added that sensory-seeking through movement (e.g. over-exercising) and differences in executive functioning might also play a role. These findings have been supported by other qualitative research on Autism in restrictive eating [24]. However, the restrictive, over-controlled presentations described in Autism-restrictive ED models may not apply to bulimic-spectrum ED’s impulsive binge-purge cycles [25, 26].

A more recent study considered the binge eating side of this and found that possibly Autistic (and ADHD) adults with binge eating pathology reported that their Neurodivergence contributed to their eating difficulties [20]. For example, Autistic-related traits like strong food preferences and texture sensitivities sometimes led to skipped meals, which in turn led to binging [20]. Interoceptive differences, like mistaking thirst for hunger or missing fullness cues and fixating on certain foods also contributed to bingeing [20]. Participants also described bingeing to meet sensory needs [20]. However, this sample did not include those with purging or more mixed bulimic symptomatology and was a mixed clinical and community sample. Thus, findings may not be relevant for patients with bulimic-spectrum EDs.

Autistic patients also have specific treatment needs. A Delphi study with Autistic women with restrictive EDs called for Autism-informed care, including clear communication, sensory-adapted environments, and staff with Autism lived experience or expertise [27]. Participants emphasised the need to distinguish Autistic traits from ED symptoms and supported adaptions like the PEACE pathway (Pathway for Eating disorders and Autism developed from Clinical Experience; https://www.peacepathway.org/) [27], which includes staff training, communication aids, and sensory-friendly interventions [28–30]. This pathway has been associated with reduced inpatient admissions and cost savings of over £22,000 per patient [31]. However, many patients with bulimic-spectrum EDs are treated in outpatient settings, where inpatient-focused interventions like the PEACE menu may be less applicable [32]. Possibly Autistic (and ADHD) adults with binge eating pathology described wanting personalised treatment, that considered sensory and communication needs [20]. Thus, further Autism-friendly adaptions need to be developed with bulimic-spectrum presentations in mind.

### Understanding Neurodivergence

Developing self-awareness and self-understanding is a key feature of most ED treatment. Psychoeducation and therapy supports patients in identifying factors contributing to their ED (such as personality traits, coping styles, and social context). It may therefore be beneficial for patients to recognise how Neurodivergent traits may influence their EDs, to help them develop coping strategies. In a recent study on possibly ADHD and Autistic adults with binge eating pathology [20], participants without formal diagnoses described uncertainty about whether they were Neurodivergent or how this related to their eating behaviours. They often wished clinicians had helped them explore these links. Those who understood how ADHD or Autistic traits influenced their binge eating reported improved self-management but typically gained this knowledge from social media rather than clinical input [20]. This highlights a gap in psychoeducation for individuals with high ADHD or Autistic traits but no diagnosis.

ADHD and Autism are also increasingly being recognised as important aspects of identity [33–35], with some individuals embracing their Neurodivergence [34], while others experience ambivalence due to stigma [20, 36]. A positive Neurodivergent identity is linked to improved well-being and lower distress [37, 38]. Bulimic-spectrum EDs are often linked to an unstable or fragmented sense of self [39], and for many patients, identity confusion or self-worth tied narrowly to body image can perpetuate symptoms [40–42]. Fostering a clear, affirming Neurodivergent identity may therefore support recovery from an ED. However, there is also concern that replacing an ‘ED identity’ with an unexamined ‘Neurodivergent identity’ may not address these underlying identity issues. Thus, exploring patients’ understanding of their own Neurodivergence and traits remains an important, yet underexplored area of research.

### Current study

This study explored the experiences of ADHD and Autistic or potentially ADHD and Autistic adults with bulimic-spectrum EDs, focusing on understanding of Neurodivergence, perceived causal mechanisms, and treatment needs. It was guided by three research questions:

1. *Understanding Neurodivergence*—How do Neurodivergent and potentially Neurodivergent adults with bulimic-spectrum EDs make sense of their (potential) Neurodivergence?
2. *Causal mechanisms—*How do they perceive their (potential) Neurodivergence as influencing bulimic-spectrum EDs development or maintenance?
3. *Treatment needs—*What are their self-identified treatment needs due to their (potential) Neurodivergence?

This work was motivated by feedback from the PEACE pathway, highlighting gaps in ADHD and bulimic-spectrum ED care, and aligns with community-identified research priorities (understanding causal mechanisms and improving treatment outcomes) [39]. Our aim is to contribute to a more inclusive, person-centred understanding of bulimic-spectrum EDs among Neurodivergent individuals and to inform best practices for care.

## Methods

### Study design

This qualitative study used semi-structured interviews and reflexive thematic analysis to explore how ADHD or potentially ADHD adults with experiences of bulimic-spectrum EDs (with and without co-occurring Autism or potential Autism) make sense of their (potential) Neurodivergence, how it influences their experience of their ED, and what support they need. National Health Service (NHS) ethical approval was obtained (REC: 24/LO/0573).

Reflexive thematic analysis was chosen to identify patterns across a diverse sample, enabling examination of both convergent and divergent experiences in ADHD-only and ADHD + Autism groups. A critical realist framework informed by the Neurodiversity paradigm and social identity theory underpinned the work [5, 33, 43, 44], recognising traits as real but their social meanings as constructed. Language choices (e.g. ‘Autistic’, ‘Neurodivergent’) reflect neuro-affirmative principles and community preferences [45–47], but quotations retain participants’ original wording, including deficit-based terms, to reflect their perspectives. This also acknowledges that some experiences of Neurodivergence may involve intrinsic challenges [1].

### Participant recruitment

Eligible participants were aged 18 + ; with a current or previous diagnosis of bulimia nervosa, atypical bulimia nervosa, or purging disorder; and either self-reported a diagnosis of ADHD/Autism or scored above cut-off on screening tools (ASRS-Screener > 3; AQ-10 > 5). We included self-identified or high-trait individuals due to diagnostic barriers and to align with current ED service practices [32, 48, 49]. This is consistent with previous studies in this area [9]. We aimed for 12–15 participants, consistent with previous studies [23, 50, 51], to achieve diversity while allowing for depth, as saturation is not a goal of reflexive thematic analysis [52].

Fifteen participants were recruited from South London and Maudsley NHS Foundation Trust ED Outpatient Services, which has implemented the PEACE pathway. All adult South London and Maudsley NHS patients with a recorded bulimia nervosa diagnosis and who had consented to be contacted for research within the last year were invited to participate, regardless of known or suspected Neurodivergence. Because all eligible South London and Maudsley NHS patients with bulimia nervosa were contacted, we could not over-sample underrepresented groups, and it was not feasible to manually review OSFED/EDNOS cases to identify all eligible atypical bulimia nervosa or purging disorder cases. However, sometimes atypical bulimia nervosa cases had been coded as bulimia nervosa rather than ‘eating disorder not otherwise specified (EDNOS)/other specified feeding or eating disorder (OSFED)’ and purging disorder cases were sometimes additionally coded as purging disorder rather than just ‘EDNOS/OSFED’ and so were included.

Clinicians acted as gatekeepers using an opt-out approach. Two participants withdrew, leaving 12 from this route. An additional three participants joined through a concurrent survey, and one volunteered after hearing about the project. Patients received study materials via email, including the participant information sheet, consent form, and the ASRS-Screener and Glasgow sensory questionnaire (GSQ-14; not analysed here) [53].

### Data collection

Once consent form and screeners were returned, clinical data was extracted from South London and Maudsley NHS’s ED database, using assessment data where available (*n* = 12), or treatment start data (*n* = 3). This data included demographics, body mass index (BMI) data, AQ-10 scores, and self-reported Autism or ADHD diagnoses. The participant not recruited from South London and Maudsley NHS completed an equivalent questionnaire.

Interviews were conducted from February to May 2025 by LM and AM. In line with AASPIRE (Academic Autistic Spectrum Partnership in Research and Education) guidelines for Autistic inclusion [54], interviews were offered in-person, or via video call, phone, or instant messaging. All chose video (69%) or in-person (31%). At the beginning of the interview, participants were told if they screened positively on the ASRS-Screener or AQ-10, with clarification that these were not diagnostic tools. Participants could complete the interview in one or two sessions—only one person opted for two. Excluding this, interviews averaged 48 min (range = 24–59).

Participants then received a £25 voucher. One emailed a post-interview reflection, which was included in the dataset. All participants received a summary of findings and were invited to share feedback. Five (31%) responded, and feedback is summarised under *member reflections* in findings*.*

### Measures

#### Adult ADHD self-report screener scale (ASRS-Screener)

Used to screen for high ADHD traits. It includes six questions predictive of DSM-IV-TR (Diagnostic and Statistical Manual of Mental Disorders, 4th Edition, Text Revision) criteria [55]. For the first three questions, responses ‘never’ and ‘rarely’ are scored 0 and ‘sometimes’, ‘often’, or ‘very often’ are score 1. For the last three questions, ‘sometimes’ is scored as 0. A score of > 3 indicates probable ADHD. It has demonstrated reliability in ED populations [56] and is widely used [8, 11, 57–60]. In the current study, internal consistency was questionable (*a* = 0.60), and all participants scored above threshold. This may reflect sample heterogeneity, screener brevity, false positives, or sample bias (as ADHD was mentioned in study invites).

#### Autism spectrum quotient—10 items (AQ-10)

Used to screen for high Autistic traits [61] and extracted from the ED service’s clinical records. It consists of 10 items scored 0–1, with a clinical cut-off of > 5. Though widely used in ED research, its reliability in acute settings is debated due to low specificity (28%) and internal consistency (α = 0.64) [62, 63]. Still, it is the only Autism screener currently recommended by NICE (National Institute for Health and Care Excellence) for adult clinical use. In the current study, internal consistency was similarly low (*a* = 0.65).

#### Interview schedule

The interview was developed from previous research [10, 23, 50, 51] and covered ED development/maintenance and treatment experiences, referring explicitly to ADHD and Autism. It was reviewed by KT, an experienced clinician. After 12 interviews, the schedule was revised. Questions that did not elicit relevant data were removed and additional questions based on findings so far were added.

### Data analysis

Interviews were audio-recorded and transcribed—some auto-transcribed and checked, others manually transcribed verbatim. All were anonymised. Full transcripts are available on request, including for secondary analysis.

We conducted reflexive thematic analysis to explore participants’ understanding of their Neurodivergence and ED, including identity and support needs. LM and AM read each transcript, made reflective notes, and discussed early impressions. LM led coding, using AM as a *critical friend* to challenge assumptions and strengthen interpretation [58]. Initial codes were developed in Word, then organised into candidate themes. Participants were then divided into five sub-groups, based on whether their ADHD was diagnosed or traits, and whether they also had an Autism diagnosis, traits, or not. LM then re-analysed these in NVivo 14. Thus, group-specific patterns could be explored and integrated into a refined thematic structure, representing both shared and divergent experiences.

Themes were then shared with participants for *member reflections* [64]. This was especially valuable in addressing the double empathy problem—mutual misunderstandings that can arise between Neurodivergent and non-Neurodivergent individuals due to differing ways of experiencing the world [65]. Feedback was used to enhance analysis, rather than to ‘validate’ findings [65]. No participants reported feeling misrepresented.

### Positionality and reflexivity

LM is a PhD student in ED and Neurodivergence research, with a background in experimental psychology. Her approach developed toward a critical realist perspective during the project. AM is a clinician in a group-based ED programme with training in social psychology and therapy. Her clinical background helped ground interpretations in clinical practice and informed ethical considerations, including the avoidance of dual roles. We chose not to disclose fixed identities but privately reflected on how varying degrees of shared experience with participants shaped our interpretations.

We were also guided by previously identified lived experience priorities [66], incorporated extended participants quotes to preserve authenticity of voices, sought feedback on findings to enhance inclusivity, followed AASPIRE’s inclusive research guidelines [54], and framed the study within a critical Neurodiversity paradigm aimed at improving ED care for ADHD and Autistic individuals.

## Results

### Sample characteristics

Sixteen adults (aged 18–52 years; *M* = 30) participated. Most were female (*n* = 13, 81%), white (*n* = 13, 81%), and heterosexual (*n* = 11, 69%). At the point of admission to the service, 14 had bulimia nervosa diagnoses, one had atypical bulimia nervosa, and one had purging disorder. Two fluctuated between anorexia nervosa (binge-purge subtype) and bulimia nervosa (one currently had bulimia, the other had just passed under the weight threshold into anorexia nervosa). BMI (body mass index) data was available for nine participants and ranged from ‘underweight’ to ‘severely obese’ (16.8–40.3). Most participants had received cognitive behavioural therapy (CBT), primarily guided self-help (GSH) or condensed self-guided (CBT-T). Others had tried enhanced CBT (CBT-E), dialectical behavioural therapy (DBT), schema therapy, compassion-focused therapy, hypnotherapy, family-based therapies, or counselling.

Six reported ADHD diagnoses, two Autism diagnoses, and one Dyslexia. Five reported taking stimulant medications. Additionally, five reported that they suspected they had ADHD and two reported suspecting Autism. All screened above threshold on the ASRS-Screener (M = 4.7), and ten also scored above threshold on the AQ-10 (M = 5.4). Twelve reported depression, eleven anxiety, three personality disorders, three post-traumatic stress disorder (PTSD) or complex PTSD, and one panic disorder. Physical health conditions included Ehlers-Danlos, endometriosis, gastroparesis, and irritable bowel syndrome (IBS).

### Reflexive thematic analysis findings

**Fig. 1:**
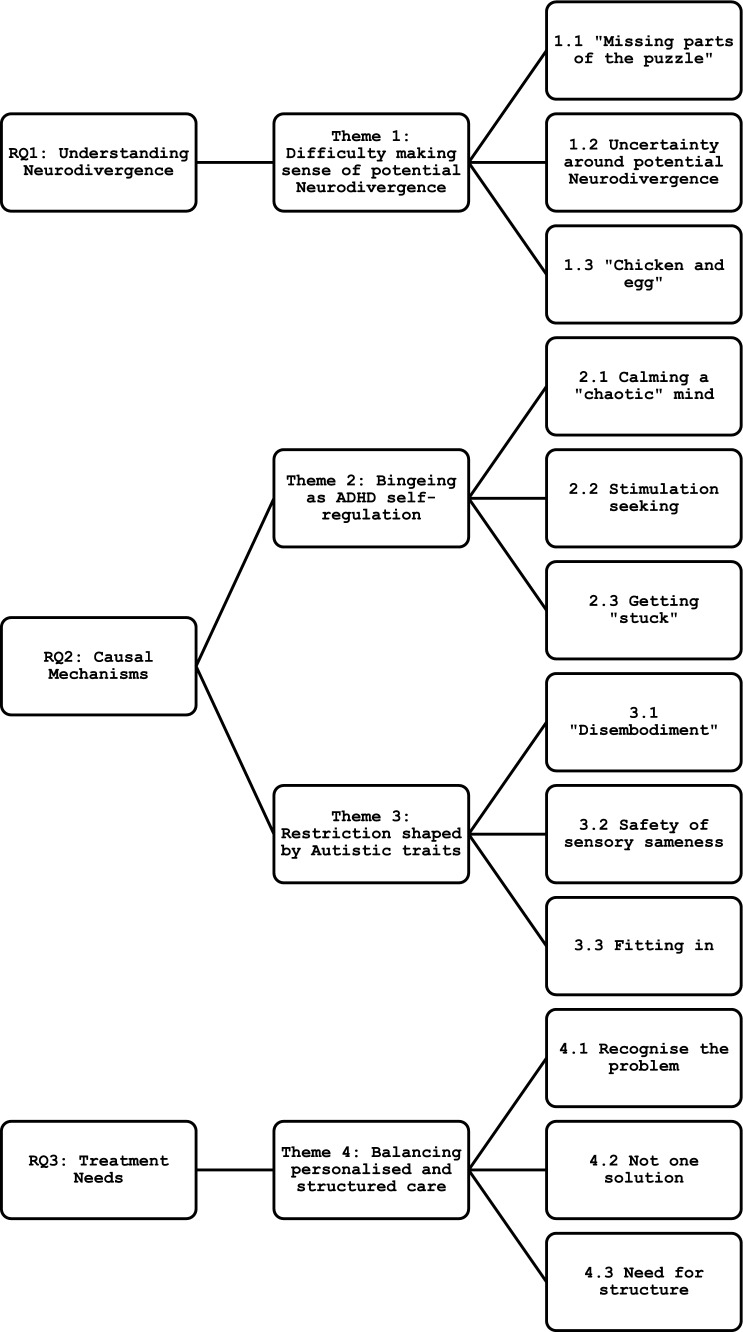
Structure of themes and sub-themes, aligned with research questions (RQs)

Participants in this study discussed their experiences of Neurodivergence or potential Neurodivergence, their ED, and treatment. Four main themes were generated (see Fig. 1).

### Theme 1: Difficulty making sense of potential Neurodivergence

This theme explores how participants understand their Neurodivergence or potential Neurodivergence, and its relationship to their ED.

#### “Missing parts of the puzzle”

Both formally diagnosed and self-identified Neurodivergent participants felt *“relief”* [006] in attributing long-standing challenges to Neurodivergence. Thus, these identities helped contextualize the onset of their ED, with Neurodivergence as the missing *“parts of the puzzle”* [003].

#### Uncertainty around potential Neurodivergence

Some without diagnoses felt unsure or conflicted about whether or not they were Neurodivergent, e.g. “*I don't have ADHD. I don't know. Well, I do have ADHD”* [016]. This uncertainty was sometimes linked to ED clinicians raising the possibility of Neurodivergence without a subsequent referral or assessment and from patient’s not understanding how Neurodivergence might *“manifest itself in different people”* [005]. This ambiguity complicated their understanding of their symptoms and responses to treatment.

#### “Chicken and egg”

Participants struggled to distinguish ED symptoms from Neurodivergent traits, due to overlapping traits, e.g. *“the patterns that you have with ADHD they can get mirrored with an eating disorder”* [012]. ED behaviours were often seen as both coping mechanisms for, and exacerbated by, neurocognitive differences, especially in ADHD, i.e. Neurodivergent traits *“fuel the fire”* of the ED [012].

Overall, self-understanding and identity formation was complicated in this sample by a lack of understanding over whether they themselves were Neurodivergent, what Neurodivergence might look like for them, and how it may be impacting their ED.

### Theme 2: Bingeing as ADHD self-regulation

This theme explores how participants felt ADHD-related experiences—such as emotional intensity, under-stimulation, and attentional differences—influenced their ED.

#### Calming a “chaotic” mind

Participants described feeling *“emotionally volatile”* [008], *“dysregulated”* [029], and *“overwhelmed”* [013] due to ADHD, and triggered by stress, change, and tasks like shopping or cooking, often leading to avoidance. Restriction provided a feeling of *“control*” [017], while bingeing provided relief, with food used to *“comfort”* [006]*, “distract”* [006]*,* or *“re-regulate*” [029]. Food often *“needed to feel substantial*” [017] for these effects. These behaviours linked to ADHD traits like impulsivity and demand avoidance, and to a lesser extent to Autism-related self-soothing. ADHD-related “*scatterbrained”* [008] thinking hindered alternative coping, and participants were sometimes resistant to structured meal planning as it removed the emotional connection to food. However, some participants lost interest in food when stressed.

#### Stimulation seeking

Bingeing or snacking also served to relieve boredom or avoid tasks. Food became a source of stimulation, “*procrastination”* [028], or a “*dopamine hit”* [029], and purging was described as energizing or motivating due to a post-behaviour *“adrenaline spike”* [007]. Novelty or engaging distractions could sometimes prevent binge-purge episodes. Binging or purging were also used to seek physical sensory experiences (e.g. “*big chunks [of food] that I can swallow […] so that I can feel my throat when they go down”*) [007].

#### Getting “stuck”

Participants described *fixating”* [012] or getting *“stuck”* [013] on food and related behaviours, often automatically or compulsively, driven by ADHD-related tendencies toward addiction or hyperfocus. Food sometimes replaced lost interests or served as a consistent source of enjoyment. Even when eating patterns began as functional, they evolved into rigid, repetitive behaviours. Special interests also fed into this theme, and EDs were seen as a *“socially acceptable”* [029] special interest for women.

*“I think for ADHD people there's a very huge connection to obviously addictive behaviours [...] food has that kind of quality or role in their lives where they just kind of look for that next excitement.”* [007]

Overall, bingeing and other ED behaviours were used to navigate ADHD-related differences, but participants struggled to disengage from these behaviours even after they stopped serving a purpose.

### Theme 3: Restriction shaped by Autistic traits

This theme explores how participants felt Autism-related experiences—such as sensory processing differences, preference for sameness, and social disconnection—influenced their ED.

#### “Disembodiment”

Autistic participants reported long-term low interoceptive awareness (e.g. not recognizing hunger, thirst, or temperature changes), often with emotional numbness. Yoga and emotion wheels were both suggested as methods to combat this. This led to unstructured or irregular mealtimes, exacerbating restriction and contributing to eventual overeating. Misinterpretation of internal sensations also sometimes triggered purging.

*“I won't realize I'm hot or cold or hungry or like thirsty in particular, until I'm like desperate […] I would have gaps between eating, which meant that when I got really, really hungry I would like overeat”* [008]

ADHD-only participants experienced similarly reduced hunger cues, but these were more often milder or episodic and linked to executive functioning and attentional differences like hyperfocus, time-blindness, or memory issues.

#### Safety of sensory sameness

Autistic participants often ate a small range of foods due to long-standing sensory sensitivities or “*preferences for sameness*” (or “*rigidity”*) [003]. Gastrointestinal issues and “*intrusive thoughts about food*” [006] also contributed to this. Participants often followed strict eating routines—specific textures, temperatures, preparation methods, or set mealtimes—where disruptions could trigger bingeing. These needs often conflicted with treatment expectations. In ADHD-only participants, preferences for sameness or sensory sensitivities often faded after childhood or had little impact on food variety or ED behaviours.

#### Fitting in

Across the sample, body image concerns and social pressure initiated restrictive behaviours. However, Autistic participants additionally described ongoing social challenges that contributed to their ED, including feeling different, withdrawing from social situations, and finding it difficult to eat around others due to pressure to mask (e.g. “*I like eating certain parts of things separately. […] Whereas if I'm in a social setting, I have to […] be more normal with things I guess.”* [006]). Whereas ADHD-only participants more often reported discomfort in specific settings, such as canteens. The ED and restriction also sometimes served as expressions of distress when verbal communication failed (e.g. *“attention seeking”* [012]) and was also linked to social exclusion and minority stress from intersecting identities, including but not limited to Neurodivergence.

Overall, Autism-related sensory differences (interoceptive awareness and exteroceptive sensory sensitivities) and social alienation often exacerbated restriction and other ED behaviours.

### Theme 4: Balancing personalised and structured care

This theme explores participants’ preferences for ED treatment.

#### Recognise the problem

Participants wanted clinicians to recognise and understand Neurodivergence and avoid pathologising difference. They emphasised ADHD involves more than inattentiveness (e.g. *“[ADHD] is so different in so many people”* [012], *“for me, definitely emotional dysregulation is a big thing”* [017]), and advocated for early screening and better-informed medication support.

#### Not one solution

Rather than set adaptions, participants stressed the need for flexible, collaborative care tailored to individual strengths and needs. These had to be developed collaboratively, as participants were often unsure of their needs or available options. They valued clinician flexibility, trust-building, and room for off-topic discussion.

*“There’s still a very much strong degree of myself trying to cope with the ADHD so […] I don't really know what adaptations would have worked but I feel like there definitely is requirement for that.”* [017]

Suggested adaptations included flexible scheduling for ADHD, allowing fidget toys and breaks, and case-by-case medication review (*“I’ll constantly want to fidget. I want to get up, I want to do something”* [024]). Autistic participants noted therapy tasks and measures could be challenging due to preferences for concrete thinking and difficulty processing abstract concepts.

#### Need for structure

Despite valuing personalisation, participants also needed consistency and clear routines, “*like if they were like reminders that are being sent, making sure that they're sent at the same time 'cause the one time that didn't happen, I freaked out*” [005]. Structured treatment, reminders, and environmental predictability supported recovery, particularly in early stages. External scaffolding (e.g., meal reminders, fixed therapy rooms) was critical, *“she encouraged me to eat like once and every two to three hours. And it kind of worked out perfectly with my job because I get like 3 breaks a day.”* [008].

Overall, participants want Neurodivergence understood in its complexity during treatment, with personalised ED care that remains structured and consistent.

### Member reflections

Participants described the research as positive, meaningful, and validating. Many felt the findings resonated with their experiences and helped them better understand their identities, such as distinctions between ADHD and Autism. While not all findings reflected individual experiences, this was seen as a strength by participants, highlighting the diversity of Neurodivergent experiences. Participants valued contributing to work that they felt was important and that could improve future treatment and all expressed interest in future research. Some found discussing EDs challenging but therapeutic and suggestions for improvement included clearer interview structure and more accessible formats for feedback. Use of member reflections was widely appreciated and encouraged for future studies.

## Discussion

Our findings show how patients understood their experiences of bulimic-spectrum EDs in relation to Neurodivergence. Despite voicing uncertainty about their (potential) Neurodivergence and how this intersected with their ED, participants described clear links between specific Neurodivergent traits and ED behaviours. Bingeing was commonly associated with ADHD-related traits, including emotional intensity, under-stimulation, and sensory or attentional drives. In contrast, restrictive behaviours were more often attributed to Autism-related traits such as sensory processing differences and social disconnect. Participants reported a desire for Neurodivergent-informed care that balances personalisation with predictable structure.

### Theoretical implications

This study offers a qualitative lens on how Neurodivergent traits interact with bulimic-spectrum EDs, expanding theoretical models focused on Autism and AN [10–12] and corroborating findings from adults with binge eating pathology [20]. Our findings demonstrate the transferability of these models to bulimic-spectrum EDs, with participants describing similar experiences underlying the connection between Autism-related traits (such as sensory processing and social differences) and restrictive or irregular eating [10, 23, 50].

However, rather than mapping specific traits to specific ED behaviours, participants described more complex, overlapping mechanisms. For instance, interoceptive difficulties contributed to both under- and overeating, as has been described elsewhere [20, 51]. Similarly, although ADHD-related traits were mostly associated with binging, some participants described restrictive behaviours resulting from hyperfocus or avoidance of overstimulating environments. Furthermore, sensory and social differences appeared in both ADHD and ADHD + Autism presentations, as has been seen elsewhere [67–69]. These overlaps highlight the need to move beyond rigid diagnostic boundaries and caveat our simplification of ‘Autism leads to restriction’ and ‘ADHD leads to bingeing’, reminding us all to attend to the lived complexity of Neurodivergent experiences in EDs.

**Fig. 2:**
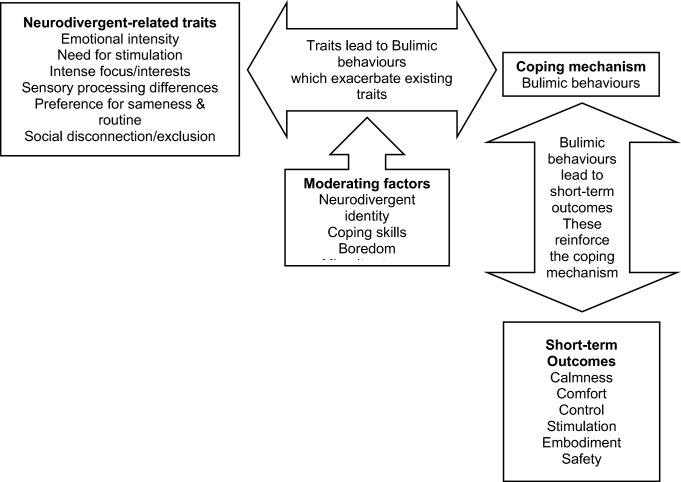
Proposed model of ADHD- and Autism-specific mechanisms underlying ED behaviours in adult patients with bulimic-spectrum EDs

This is also the first study looking at qualitative experiences of ADHD and potentially ADHD adults with bulimic-spectrum EDs. While literature often links ADHD and binge eating through impulsivity, reward processing, and emotional intensity [15–18], these concepts only partially captured participants’ experiences. Impulsivity subtypes like sensation seeking or negative urgency [70] were more relevant, especially where bingeing was used to manage emotional intensity. Executive function challenges—such as memory lapses or poor planning—also contributed to chaotic eating. This aligns with previous findings in possibly ADHD (and Autistic) adults with binge eating pathology [20]. Our sub-theme ‘getting stuck’ also mirrors addiction models of bulimia nervosa, where impulsivity shifts to compulsion [71]. These findings can build an expanded model of Neurodivergence’s influence on bulimic-spectrum EDs (see Fig. 2).

Future studies could validate and quantify the role of these mechanisms (e.g. using multi-dimensional impulsivity measures like the UPPS Impulsive Behaviour Scale [72]), investigate how these mechanisms may vary across individuals (e.g. using network analysis to identify distinct presentations of ADHD in ED populations, as has been done in neurotypical ED populations [73]), and how these mechanisms may influence each other (e.g. increased social isolation has been linked to heightened hyper-focus in Neurodivergent adults [74] and to altered eating behaviours, including cravings, reward-based eating, loss of control, and food addiction [75]).

Notably, purging was rarely discussed and, when mentioned, was typically framed as secondary to bingeing rather than directly linked to Neurodivergence. Occasional exceptions included using purging to release endorphins to overcome procrastination or in response to misinterpreted bodily signals. This may reflect a reduced association between purging and Neurodivergence, aligning with previous findings that ADHD more closely links to binge eating symptoms than bulimic symptoms [12]. It could also result from underreporting due to stigma.

### Clinical implications

Many participants were unclear about if they were Neurodivergent or not and did not understand how it related to their ED. Our findings highlight an urgent need for ED services to better identify and support Neurodivergent patients and those with high Neurodivergent traits. Treatment should offer space to explore the interplay between Neurodivergent traits and ED symptoms.

With greater understanding of an individual’s Neurodivergent traits, treatment can offer more targeted strategies. For emotional intensity, treatment may focus on acceptance-based CBT skills (shown to reduce negative urgency in patients with binge-type EDs [73]), emotion wheels, structured meals, and alternative self-soothing strategies; for sensory needs, options like sensory workshops, do-it-yourself sensory toolkits, and interoception-based interventions like ADIE (Aligning Dimensions of Interoceptive Experience; improves interoceptive awareness and reduces anxiety in Autistic adults [76]); for social disconnection, identity-affirming and peer-support approaches may be needed; and for addiction-like patterns, pharmacological options (e.g. bupropion, naltrexone are effective for food addiction [77]; other medications for substance-use disorders in ADHD populations [72]) and trauma-informed approaches (recommended by Autistic adults for substance-use treatment [78]).

Participants consistently emphasised the need for personalised, compassionate care that validates Neurodivergent experiences. Prior studies have noted that Neurodivergent patients often feel misunderstood or harmed by generic CBT models and other psychological models developed for neurotypical patient groups [79]. Our findings reinforce the need for services to offer tailored psychological interventions. Clinicians could benefit from more training and support in recognising how Neurodivergent traits affect treatment engagement and adapting appropriately (e.g. allowing fidget toys, offering breaks, avoiding abstract tasks or confusing metaphors), as recommended in other contexts [78]. Initiatives like the PEACE Pathway (https://www.peacepathway.org/) developed primarily for Autism and AN already incorporate strategies like communication passports and sensory adaptations [28–30] which may be equally beneficial for ADHD and bulimic-spectrum ED populations. However, ADHD-specific adaptions are still underdeveloped.

Future research studies should explore factors influencing Neurodivergent self-identification and clinician recognition in this population. Measures like the Autistic Identity Questionnaire (AIQ) may support this [80].

### Strengths and limitations

This study explores how ADHD (and co-occurring Autism) interact with bulimic-spectrum EDs, adding patient perspectives (particularly underexplored in ADHD) and addressing self-understanding and identity for the first time in this group. Inclusive recruitment captured diagnosed and high-scoring participants, with accessible interview formats. Rigour was strengthened through critical friend discussions and member reflections addressed the double empathy problem. Conducted in a real-world NHS service, findings offer clinically relevant, actionable insights for personalising treatment and improving engagement in Neurodivergent populations.

Overall, our sample was highly heterogeneous: possible Neurodivergence was defined by diagnosis, self-identification, or high traits, and participants had a range of bulimic-spectrum EDs and were at various stages of treatment and recovery. Although this complicates the analysis, it also enriches it by allowing for a more nuanced understanding of the subject matter. Participants had varying ED behaviours and varying levels of self-awareness regarding their Neurodivergent traits and ED. This added variety to the data while also adding limitations.

Limitations include the potential dilution of findings by including undiagnosed participants and the absence of an Autism-only group, although this reflects clinical reality (Autism-only presentations are rarer in bulimic-spectrum EDs than in restrictive EDs [7]). BMI data was limited in our sample as patients have to self-report this in the ED service’s database. Demographic diversity was also limited in our sample, and future studies should prioritise underrepresented groups and explore other bulimic-spectrum diagnoses (e.g., anorexia nervosa binge-purge subtypes) where Autism traits may be more pronounced. Further research could also apply alternative qualitative methods to either narrow down (e.g. Interpretive Phenomenological Analysis to gain deeper insight into the lived experience of ADHD in the context of bulimic-spectrum EDs) or widen focus (e.g. grounded theory to develop a more comprehensive model of how Neurodivergence shapes ED experiences).

## What is already known on this topic?

Previous research has explored how Autistic individuals with restrictive EDs understand the role of their Neurodivergence in their ED, but it's unclear how those who do not identify as Neurodivergent, or those with bulimic-spectrum EDs, perceive these links, limiting transferability. Similarly, while theoretical models connect ADHD to binge eating, few studies have examined how ADHD individuals personally experience or understand these connections, reducing their practical value for treatment and self-understanding. Lastly, little was known about what ADHD patients want from ED treatment.

## What this study adds to this topic?

This study shows that much of our existing understanding of how Autistic traits contribute to anorexia nervosa can also be used to understand how they contribute to bulimic-spectrum EDs. However, many patients with Autistic traits may be unaware of how these may be influencing their bulimic behaviours. This study also adds patient-informed connections between ADHD traits and bulimic behaviours, improving their relevance for both patients and clinicians. Personalised care could be improved by exploring these Neurodivergent-ED links while maintaining the structure and routine that many patients find supportive.

## Conclusions

ED services may be improved by supporting both patients and clinicians in recognising and understanding Neurodivergent traits. Clinicians may benefit from training on how ADHD traits, such as emotional overwhelm, under-stimulation, sensory seeking, and addiction-like tendencies can contribute to bingeing; and how Autistic traits such as interoceptive difficulties, exteroceptive sensory sensitivities, and social difficulties can contribute to restriction. Treatment could be adapted for these mechanisms and balance personalised, flexible approaches with the stabilising benefits of structure and routine.

## Data Availability

The datasets used during the current study are available from the corresponding author on reasonable request, and can be used for secondary analysis as is stipulated in participants’ consent forms.

## References

[1] Botha M, Gillespie-Lynch K (2022) Come as you are: examining autistic identity development and the neurodiversity movement through an intersectional lens. Hum Dev 66(2):93–112

[2] Pellicano E, den Houting J (2022) Annual research review: shifting from ‘normal science’to neurodiversity in autism science. J Child Psychol Psychiatry 63(4):381–396 34730840 10.1111/jcpp.13534 PMC9298391

[3] APA (2013) Diagnostic and statistical manual of mental disorders: DSM-5. American psychiatric association, Washington, DC

[4] Antshel KM, Russo N (2019) Autism spectrum disorders and ADHD: Overlapping phenomenology, diagnostic issues, and treatment considerations. Curr Psychiatry Rep 21:1–11 30903299 10.1007/s11920-019-1020-5

[5] Beck TJ (2024) Neurodivergent culture and embodied knowledge beyond neoliberal identity politics. Cult Psychol 30(3):736–756

[6] Cobbaert L et al (2024) Neurodivergence, intersectionality, and eating disorders: a lived experience-led narrative review. J Eat Disord 12(1):187 39568093 10.1186/s40337-024-01126-5 PMC11580580

[7] Makin L et al (2025) Autism, ADHD, and their traits in adults with bulimia nervosa and binge eating disorder: a scoping review. Eur Eat Disord Rev. 10.1002/erv.3177 39865514 10.1002/erv.3177 PMC12171673

[8] Svedlund NE et al (2017) Symptoms of attention deficit hyperactivity disorder (ADHD) among adult eating disorder patients. BMC Psychiatry 17:1–9 28095885 10.1186/s12888-016-1093-1 PMC5240294

[9] Nimbley E et al (2024) A mixed method systematic review into the impact of ED treatment in Autistic people and those with high Autistic traits. Int J Eat Disord. 10.1002/eat.24311 39541220 10.1002/eat.24311 PMC11784838

[10] Brede J et al (2020) “For me, the anorexia is just a symptom, and the cause is the autism”: investigating restrictive eating disorders in autistic women. J Autism Dev Disord 50:4280–4296 32274604 10.1007/s10803-020-04479-3 PMC7677288

[11] Testa G et al (2020) Does ADHD symptomatology influence treatment outcome and dropout risk in eating disorders? A longitudinal study. J Clin Med 9(7):2305 32698514 10.3390/jcm9072305 PMC7408799

[12] Nazar BP et al (2016) The risk of eating disorders comorbid with attention-deficit/hyperactivity disorder: a systematic review and meta-analysis. Int J Eat Disord 49(12):1045–1057 27859581 10.1002/eat.22643

[13] Kessler RC et al (2013) The prevalence and correlates of binge eating disorder in the World Health Organization World Mental Health Surveys. Biol Psychiatry 73(9):904–914 23290497 10.1016/j.biopsych.2012.11.020 PMC3628997

[14] Fayyad J et al (2017) The descriptive epidemiology of DSM-IV adult ADHD in the world health organization world mental health surveys. ADHD Atten Deficit Hyperact Disord 9(1):47–65 10.1007/s12402-016-0208-3 PMC5325787 27866355

[15] Cortese S, Bernardina BD, Mouren M-C (2007) Attention-deficit/hyperactivity disorder (ADHD) and binge eating. Nutr Rev 65(9):404–411 17958207 10.1111/j.1753-4887.2007.tb00318.x

[16] Reinblatt SP (2015) Are eating disorders related to attention deficit/hyperactivity disorder? Curr Treat Options Psychiatry 2:402–412 26949595 10.1007/s40501-015-0060-7 PMC4777329

[17] Seymour KE et al (2015) Overlapping neurobehavioral circuits in ADHD, obesity, and binge eating: evidence from neuroimaging research. CNS Spectr 20(4):401–411 26098969 10.1017/S1092852915000383 PMC4560968

[18] El Archi S et al (2020) Negative affectivity and emotion dysregulation as mediators between ADHD and disordered eating: a systematic review. Nutrients 12(11):3292 33121125 10.3390/nu12113292 PMC7693832

[19] Barrios L et al (2022) Reinforcement sensitivity and bulimia symptoms: the role of emotion regulation. Eat Weight Disord Stud Anorexia Bulimia Obes. 10.1007/s40519-021-01275-5 10.1007/s40519-021-01275-5 34546555

[20] Makin L et al. Regulating with food: a qualitative study of neurodivergent experiences in adults with binge eating disorder. Unpublished. 10.1186/s40337-025-01493-7 PMC12801551 41366481

[21] Dell’Osso L et al (2018) Subthreshold autism spectrum disorder in patients with eating disorders. Compr Psychiatry 81:66–72 29268154 10.1016/j.comppsych.2017.11.007

[22] Gesi C et al (2017) Autistic traits in patients with anorexia nervosa, bulimia nervosa or binge eating disorder: a pilot study. Eur Psychiatry 41(S1):S100–S100

[23] Kinnaird E et al (2019) Same behaviours, different reasons: what do patients with co-occurring anorexia and autism want from treatment? Int Rev Psychiatry 31(4):308–317 30821179 10.1080/09540261.2018.1531831

[24] Loomes R et al (2025) Understanding the Autistic experience of restrictive eating disorders—a systematic review and qualitative‐synthesis. Eur Eat Disord Rev. 10.1002/erv.3181 40042436 10.1002/erv.3181 PMC12171672

[25] Seitz J et al (2013) The role of impulsivity, inattention and comorbid ADHD in patients with bulimia nervosa. PLoS ONE 8(5):e63891 23700439 10.1371/journal.pone.0063891 PMC3659086

[26] Gilsbach S et al (2025) The roles of impulsivity, comorbid ADHD, and borderline personality disorder in patients with bulimia nervosa. Eat Weight Disord-Stud Anorex Bulim Obes 30(1):1–9 10.1007/s40519-025-01713-8 PMC11742761 39825963

[27] Field SL et al (2023) “Work WITH us”: a Delphi study about improving eating disorder treatment for autistic women with anorexia nervosa. J Eat Disord 11(1):17 36759874 10.1186/s40337-023-00740-z PMC9909870

[28] Tchanturia K et al (2025) Implementation insights from the PEACE pathway across UK eating disorder services. Nutrients 17(9):1532 40362840 10.3390/nu17091532 PMC12073639

[29] Li Z et al (2024) Don’t talk to me like i am an illness”: exploring patients’ needs using the communication passport in an eating disorder service. Neuropsychiatrie. 10.1007/s40211-024-00501-7 38995527 10.1007/s40211-024-00501-7 PMC11876190

[30] Li Z et al (2023) In-person and online sensory wellbeing workshop for eating disorders: updated case series. J Eat Disord 11(1):117 37443135 10.1186/s40337-023-00834-8 PMC10347786

[31] Tchanturia K et al (2021) A novel approach for autism spectrum condition patients with eating disorders: analysis of treatment cost-savings. Eur Eat Disord Rev 29(3):514–518 32648631 10.1002/erv.2760

[32] Li Z et al (2024) A qualitative evaluation of the pathway for eating disorders and autism developed from clinical experience (PEACE): clinicians’ perspective. Front Psychiatry 15:1332441 38638414 10.3389/fpsyt.2024.1332441 PMC11024361

[33] Botha M (2025) Critical realism, community psychology, and the curious case of autism: a philosophy and practice of science with social justice in mind. J Community Psychol 53(1):e22764 34897720 10.1002/jcop.22764

[34] Davies J et al (2024) Autistic identity: a systematic review of quantitative research. Autism Res 17(5):874–897 38334318 10.1002/aur.3105

[35] Frick MA et al (2025) ADHD and identity formation: adolescents’ experiences from the healthcare system and peer relationships. J Atten Disord 29(7):541–553 39963782 10.1177/10870547251318484 PMC11956369

[36] Gray SM et al (2024) An exploration of diagnostic identity for autistic individuals: A systematic review of existing literature. Res Autism Spectr Disord 114:102394

[37] Cooper K, Smith LG, Russell A (2017) Social identity, self-esteem, and mental health in autism. Eur J Soc Psychol 47(7):844–854

[38] Corden K, Brewer R, Cage E (2021) Personal identity after an autism diagnosis: relationships with self-esteem, mental wellbeing, and diagnostic timing. Front Psychol 12(3051):2021 10.3389/fpsyg.2021.699335 PMC8360844 34393933

[39] Wheeler HA, Adams GR, Keating L (2001) Binge eating as a means for evading identity issues: the association between an avoidance identity style and bulimic behavior. Identity Int J Theory Res 1(2):161–178

[40] Verschueren M et al (2017) Identity processes and statuses in patients with and without eating disorders. Eur Eat Disord Rev 25(1):26–35 27790863 10.1002/erv.2487

[41] Verschueren M et al (2018) Eating disorder symptomatology and identity formation in adolescence: a cross-lagged longitudinal approach. Front Psychol 9:816 29915548 10.3389/fpsyg.2018.00816 PMC5994691

[42] Verschueren M et al (2024) Identity functioning in patients with an eating disorder: developmental trajectories throughout treatment. Nutrients 16(5):591 38474720 10.3390/nu16050591 PMC10935138

[43] Le L. " I Am Human, Just Like You": What Intersectional, Neurodivergent Lived Experiences Bring to Accessibility Research. In Proceedings of the 26th International ACM SIGACCESS Conference on Computers and Accessibility. 2024.

[44] Koopmans E, Schiller DC (2022) Understanding causation in healthcare: an introduction to critical realism. Qual Health Res 32(8–9):1207–1214 35649292 10.1177/10497323221105737 PMC9350449

[45] Bottema-Beutel K et al (2021) Avoiding ableist language: suggestions for autism researchers. Autism Adulthood 3(1):18–29 36601265 10.1089/aut.2020.0014 PMC8992888

[46] Kenny L et al (2016) Which terms should be used to describe autism? Perspectives from the UK autism community. Autism 20(4):442–462 26134030 10.1177/1362361315588200

[47] Pineo E (2022) “ But the Bumpies Hurt!”: Autism and the Importance of Identity-first Language. Incl Disabil. 10.51357/id.vi2.194

[48] Ardeleanu K et al (2024) Self-identification of autism: Why some autistic adults lack a clinical diagnosis and why this matters for inclusion. Autism. 10.1177/13623613241297222 39552426 10.1177/13623613241297222

[49] Ahuvia I et al., Identifying as autistic without a formal diagnosis: who self-identifies as autistic and why? 2025.

[50] Nimbley E et al (2023) “It’s not about wanting to be thin or look small, it’s about the way it feels”: an IPA analysis of social and sensory differences in autistic and non-autistic individuals with anorexia and their parents. J Eat Disord 11(1):89 37277884 10.1186/s40337-023-00813-z PMC10243074

[51] Kinnaird E et al (2019) Eating as an autistic adult: an exploratory qualitative study. PLoS ONE 14(8):e0221937 31465510 10.1371/journal.pone.0221937 PMC6715205

[52] Braun V, Clarke V (2021) To saturate or not to saturate? Questioning data saturation as a useful concept for thematic analysis and sample-size rationales. Qual Res Sport Exerc Health 13(2):201–216

[53] Millington E, Simmons D. Development and validation of the Glasgow Sensory Questionnaire Short (GSQ-14). 2023.

[54] Nicolaidis C et al (2019) The AASPIRE practice-based guidelines for the inclusion of autistic adults in research as co-researchers and study participants. Autism 23(8):2007–2019 30939892 10.1177/1362361319830523 PMC6776684

[55] Kessler RC et al (2005) The World Health Organization Adult ADHD Self-Report Scale (ASRS): a short screening scale for use in the general population. Psychol Med 35(2):245–256 15841682 10.1017/s0033291704002892

[56] Carlucci S et al (2017) Validity and reliability of the attention deficit hyperactivity disorder self-report scale (ASRS-v1. 1) in a clinical sample with eating disorders. Eat Behav 26:148–154 28390269 10.1016/j.eatbeh.2017.03.010

[57] Svedlund NE et al (2018) Are treatment results for eating disorders affected by ADHD symptoms? A one-year follow-up of adult females. Eur Eat Disord Rev 26(4):337–345 29717794 10.1002/erv.2598

[58] Fernández-Aranda F et al (2013) ADHD symptomatology in eating disorders: a secondary psychopathological measure of severity? BMC Psychiatry 13:1–8 23758944 10.1186/1471-244X-13-166 PMC3693886

[59] Ferre F et al (2017) Influence of attention deficit hyperactivity disorder symptoms on quality of life and functionality in adults with eating disorders. Actas Esp Psiquiatr 45(3):98–107 28594055

[60] Ruiz Feliu M et al. Presence and influence of attention deficit hyperactivity disorder symptoms in adults with an eating disorder. In ANALES DEL SISTEMA SANITARIO DE NAVARRA. 2022. GOBIERNO DE NAVARRA NAVAS TOLOSA 21, PAMPLONA, 31002, SPAIN. 10.23938/ASSN.0984 PMC10114044 35037918

[61] Allison C, Auyeung B, Baron-Cohen S (2012) Toward brief “red flags” for autism screening: the short autism spectrum quotient and the short quantitative checklist in 1,000 cases and 3,000 controls. J Am Acad Child Adolesc Psychiatry 51(2):202–212 22265366 10.1016/j.jaac.2011.11.003

[62] Hudson CC et al (2024) Psychometric properties of the 10-item Autism Quotient in an acute psychiatric sample. Res Autism Spectr Disord 110:102299

[63] Ashwood KL et al (2016) Predicting the diagnosis of autism in adults using the Autism-Spectrum Quotient (AQ) questionnaire. Psychol Med 46(12):2595–2604 27353452 10.1017/S0033291716001082 PMC4988267

[64] Braun V, Clarke V (2024) A critical review of the reporting of reflexive thematic analysis in Health Promotion International. Health Promot Int 39(3):daae049 38805676 10.1093/heapro/daae049 PMC11132294

[65] Milton D, Gurbuz E, López B (2022) The ‘double empathy problem’: ten years on. Autism 26(8):1901–1903 36263746 10.1177/13623613221129123

[66] Keller J et al (2024) The overlap of disordered eating, autism and ADHD: future research priorities as identified by adults with lived experience. Lancet Psychiatry 11(12):1030–1036 39154650 10.1016/S2215-0366(24)00186-X

[67] Bruton AM et al (2025) Diminished interoceptive accuracy in attention-deficit/hyperactivity disorder: a systematic review. Psychophysiology 62(2):e14750 39905593 10.1111/psyp.14750 PMC11842156

[68] Ciccarelli J. ADHD, intuitive eating, interoceptive awareness and meaning mindset: are they interrelated? 2024.

[69] Bijlenga D et al (2017) Atypical sensory profiles as core features of adult ADHD, irrespective of autistic symptoms. Eur Psychiatry 43:51–57 28371743 10.1016/j.eurpsy.2017.02.481

[70] Zapolski TC et al (2010) Borderline personality disorder, bulimia nervosa, antisocial personality disorder, ADHD, substance use: common threads, common treatment needs, and the nature of impulsivity. Indep Pract (Lutterworth, England) 30(1):20 PMC3022439 21253443

[71] Pearson CM, Wonderlich SA, Smith GT (2015) A risk and maintenance model for bulimia nervosa: from impulsive action to compulsive behavior. Psychol Rev 122(3):516 25961467 10.1037/a0039268 PMC4486518

[72] Fluyau D, Revadigar N, Pierre CG (2021) Systematic review and meta-analysis: treatment of substance use disorder in attention deficit hyperactivity disorder. Am J Addict 30(2):110–121 33289928 10.1111/ajad.13133

[73] Wilkinson ML, Juarascio AS (2025) Heterogeneous profiles of impulsivity are associated with clinical severity and treatment outcomes among adults with binge-spectrum eating disorders. Eat Disord. 10.1080/10640266.2025.2520563 40568875 10.1080/10640266.2025.2520563 PMC12354265

[74] Folly JC, Reis LES, Roman-Ramos H. The Role of social isolation in shaping hyperfocus in adults with ADHD and/or ASD: a cross-sectional study. 2025.

[75] Zhang X et al (2024) Social isolation, brain food cue processing, eating behaviors, and mental health symptoms. JAMA Netw Open 7(4):e244855–e244855 38573637 10.1001/jamanetworkopen.2024.4855 PMC11192185

[76] Quadt L et al (2021) Interoceptive training to target anxiety in autistic adults (ADIE): a single-center, superiority randomized controlled trial. EClinicalMedicine. 10.1016/j.eclinm.2021.101042 34401684 10.1016/j.eclinm.2021.101042 PMC8350004

[77] Leary M et al (2021) Current intervention treatments for food addiction: a systematic review. Behav Sci (Basel) 11(6):80 34071059 10.3390/bs11060080 PMC8224570

[78] Munday K et al (2025) Improving substance-use services for autistic adults: insights and recommendations from autistic adults. Autism Adulthood. 10.1089/aut.2024.0213

[79] William S et al (2024) Experience of CBT in adults with ADHD: a mixed methods study. Front Psych 15:1341624 10.3389/fpsyt.2024.1341624 PMC11221408 38962060

[80] Overton GL. Understanding the self-identification of autism in adults within the UK population: development of a screening questionnaire. 2023, University of East Anglia.

